# Bone Defects in Tibia Managed by the Bifocal vs. Trifocal Bone Transport Technique: A Retrospective Comparative Study

**DOI:** 10.3389/fsurg.2022.858240

**Published:** 2022-05-19

**Authors:** Alimujiang Abulaiti, Yanshi Liu, Feiyu Cai, Kai Liu, Abulaiti Abula, Xiayimaierdan Maimaiti, Peng Ren, Aihemaitijiang Yusufu

**Affiliations:** Department of Trauma and Microreconstructive Surgery, The First Affiliated Hospital of Xinjiang Medical University, Urumqi, Xinjiang, China

**Keywords:** bifocal bone transport, bone defect, distraction osteogenesis, trifocal bone transport, reconstruction

## Abstract

**Background:**

The purpose of this study is to evaluate the clinical effectiveness and determine the differences, if any, between the trifocal bone transport (TFT) technique and the bifocal bone transport (BFT) technique in the reconstruction of long segmental tibial bone defects caused by infection using a monolateral rail external fixator.

**Methods:**

A total of 53 consecutive patients with long segmental tibial bone defects caused by infection and treated by monolateral rail external fixator in our department were retrospectively collected and analyzed from the period January 2013 to April 2019, including 39 males and 14 females with an average age of 38.8 ± 12.4 years (range 19–65 years). Out of these, 32 patients were treated by the BFT technique, and the remaining 21 patients were managed by the TFT technique. The demographic data, operation duration (OD), docking time (DT), external fixation time (EFT), and external fixation index (EFI) were documented and analyzed. Difficulties that occur during the treatment were classified according to Paley. The clinical outcomes were evaluated by following the Association for the Study and Application of the Method of Ilizarov (ASAMI) criteria at the last clinical visit.

**Results:**

All patients achieved an infection-free union finally, and there was no significant difference between the two groups in terms of demographic data and both ASAMI bone and functional scores (*p* > 0.05). The mean defect size and OD in TFT (9.4 ± 1.5 cm, 161.9 ± 8.9 min) were larger than that in BFT (7.8 ± 1.8 cm, 122.5 ± 11.2 min) (*p* < 0.05). The mean DT, EFT, and EFI in TFT (65.9 ± 10.8 days, 328.0 ± 57.2 days, 34.8 ± 2.1 days/cm) were all less than those in BFT (96.8 ± 22.6 days, 474.5 ± 103.2 days, 60.8 ± 1.9 days/cm) (*p* < 0.05). Difficulties and complications were more prevalent in the BFT group than in the TFT group (*p* < 0.05).

**Conclusion:**

Both the trifocal and BFT techniques achieve satisfactory clinical outcomes in the reconstruction of long segmental tibial bone defects caused by infection using a monolateral rail external fixator. The TFT technique can significantly decrease the DT, EFT, EFI, difficulties, and complications compared with the BFT technique.

## Introduction

Long segmental tibial bone defects, a common clinical problem, can result from high-energy injury, the radical removal of contaminated bony fragments in an open fracture, a bone tumor resection, or a repeated debridement of infected non-union (1, 2). The management of this complex problem is a challenge for the treating surgeons and patients, combined with exhausting and prolonged procedures, as well as various complications (3–5). Different technical options have been proposed to reconstruct bone defects, including acute shortening, vascularized or non-vascularized autografts, allografts, bone substitutes, and the Masquelet technique (6–13). However, the long duration of restricted weight-bearing, the limited source of autogenous grafts for large defects, infection, and uncertain union rates remain major concerns.

The treatment of bone defects has been revolutionized by the bone transport technique as seen by its worldwide use. A lot of published data have declared that this distraction osteogenesis (DO) technique is an effective and practical method for the reconstruction of large bone defects due to the complete eradication of infection and its powerful effects (14–20). The traditionally used bifocal bone transport (BFT) technique is a single-level transport with one osteotomy site, with the main drawbacks of long frame duration, unsatisfactory regenerates, and increasing complication incidence (17). The concept of trifocal bone transport (TFT), which is regarded as a double-level bone transport with two osteotomy sites, has been proposed to shorten the treatment duration for faster regeneration (17, 21–23). Although satisfactory clinical outcomes have been reported by many previous studies, there are only limited data focusing on a comparison of final clinical results and differences between the two techniques.

Therefore, the purpose of this study is to evaluate the clinical effectiveness and determine the differences, if any, between the trifocal and the BFT techniques in the reconstruction of long segmental tibial bone defects caused by infection using a monolateral rail external fixator.

## Patients and Methods

A total of 62 consecutive patients with long segmental tibial bone defects caused by infection and managed by the bone transport technique with a monolateral rail external fixator (Limb Reconstruction System, LRS, Orthofix, Verona, Italy) in our department were retrospectively collected and analyzed from the January 2013 to April 2019 period. The inclusion criteria were as follows: patients with tibial bone defects more than or equal to 6 cm treated by the trifocal and BFT techniques, age older than 18 years, with a 24-month minimum follow-up. We excluded patients with pathological fracture, age older than 65 years, vascular and nerve injury, bone tumor, poor compliance, smoking habit, any other illness that can affect bone healing (such as diabetes), and those managed by a circular external fixator.

After the application of exclusion criteria, 53 patients were rendered eligible for this study, including their 53 injured limbs (left limb in 22 and right limb in 31). There were 39 males and 14 females with an average age of 38.8 ± 12.4 years (range 19–65 years). Thirty-two patients were treated by the BFT, and the remaining 21 patients were managed by the TFT. The etiology of bone defects was chronic osteomyelitis in 36 patients and infected non-union in 17 patients. With regard to bone defect location, there were 8 cases in the proximal one-third of the tibial shaft, 29 cases in the middle, and 16 cases in the distal. The mean previous operation was 2.6 ± 1.0 times (range 1–5 times). A meticulous debridement of the affected tissues, the installation of a monolateral rail external fixator, and a percutaneous minimally invasive cortical osteotomy using the Gigli saw were performed in all the patients. The average bone defect size after radical debridement was 8.5 ± 1.9 cm (range 6–13 cm) measured intraoperatively. There were 11 injured limbs that suffered active infection with drainage and sinus. Samples of the infected tissues were cultured, and antibiotic susceptibility tests were conducted in each patient. The culturing results showed *Staphylococcus aureus* in 25 cases, Methicillin-resistant *Staphylococcus aureus* in 17, *Pseudomonas aeruginosa* in 7, and *Escherichia coli* in 4.

The demographic data, operation duration (OD), docking time (DT), external fixation time (EFT), and external fixation index (EFI) were collected and analyzed. All patients were followed up at a minimum of 2 years after frame removal, and none was lost. The clinical outcomes were evaluated by following the Association for the Study and Application of the Method of Ilizarov (ASAMI) criteria (24) at the last follow-up. Difficulties that occurred during the treatment were classified and documented according to Paley (25). Informed consent was obtained from all patients for their data to be documented and published in our study. The Ethical Committee of our institution approved this study.

### Surgical Procedures

The same team performed all surgical procedures. The patients were positioned supine on a radiolucent table under continuous general or regional anesthesia. The incision was carried down to the periosteum corresponding to the previous surgical incisions when possible, protecting the healthy skin or subcutaneous tissues. The infected and devitalized bone and soft tissues were radically resected after complete hardware removal, and the samples were sent to culture for a sensitive antibiotic examination. The bony ends were resected until cortical bleeding, called the paprika sign (26), which manifests in healthy osseous tissue. Frequent alternating irrigation with hydrogen peroxide, physiological saline, and iodine liquid during and after debridement is critical.

Antibiotic-impregnated cement beads were applied for patients with severe infection. Meticulous hemostasis was performed before wound closure. The vacuum sealing drainage technique was used for those with soft tissue defects or those that could not be initially closed without tension. Subsequently, local tissue flap or direct suture without tension was performed to reconstruct the small soft tissue defects, whereas flap transfer or free skin grafting was used to cover the larger wound.

**Figure 1:**
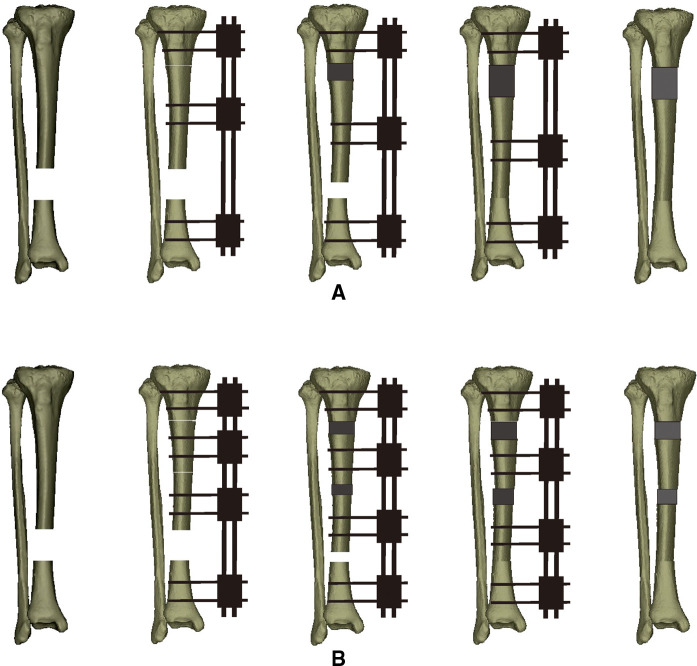
Schematic diagram of the two bone transport techniques for the management of the lower third bony segmental defects in the tibia (from left to right). (**A**) Bifocal bone transport technique. (**B**) Trifocal bone transport technique.

Length and axis restoration of the injured extremity was achieved first when the monolateral external fixator was installed. Two or three Schanz screws fixed by the connecting rail were inserted into the proximal and distal bony ends, respectively. For the BFT, the other two screws were inserted into the planned transport bony fragment and fixated at the sliding block. Every screw needed to be on the same plane. According to the location of the bone defect, a percutaneous cortical osteotomy with minimal invasion was conducted using Gigli saw. The framework of the TFT was similar but with two intermediate transport fragments fixated by the corresponding sliding blocks, as well as two osteotomy sites at the appropriate site. Bone defects larger than 8 cm or those that exceeded 40% of the injured bone underwent a TFT procedure (21, 27). When the bone defect was located at the upper or lower third tibial shaft, two osteotomies were conducted in the longer bony segment (tandem transport). One osteotomy was performed on each side of the defect when the patient suffered a central defect (converging transport). The detailed manipulations are shown in Figure 1.

### Postoperative Management

Sensitive antibiotic therapy was performed until the infection had been resolved, depending on the clinical manifestations and laboratory indicators. The small soft tissue defects were treated by direct suture or local tissue flap. The larger soft tissue defects were managed by free skin grafting or flap transfer.

All patients were encouraged to undergo the isometric muscle and joint range of motion exercise on the postoperative second day, and early full-weight-bearing was also encouraged. The foot was kept in a neutral position using a rigid shoe with an elastic band, preventing ankle equinus contracture. Regular pin site care was performed every day using medical alcohol or iodophors.

After a 7-day latency period, bone transport started at a rate of 0.25 mm in the BFT group, 4 times per day. In the TFT group, the bony fragment near the bone defect was transported 0.5 mm 4 times per day, while the other bony fragment was transported at a rate of 0.25 mm 4 times per day (tandem transport). As for converging transport, each bony fragment on both sides of the bone defect was transported 0.25 mm 4 times per day. Notably, the transport speed was modified according to the patient’s tolerance level and the radiological quality of the regenerate. After docking, bone transport continued for 4 or 5 days to compress the docking site, and the regenerate was allowed to consolidate.

During the bone transport phase, the patients were asked to pay regular clinical visits twice a month, while it was once a month during the consolidation period. The monolateral external fixator was dynamized and followed by removal when satisfactory consolidation (three uninterrupted cortices appeared at the distraction zone) and docking site complete union were achieved on the standard anteroposterior and lateral radiographs. After frame removal, a functional brace was used to prevent refracture at the docking site or bending in the distraction zone.

### Statistical Analysis

Statistical analysis was performed with SPSS 22.0 (IBM Corp., NY, USA). Independent-sample *t*-tests were used to analyze the continuous variables, which were expressed as the mean, standard deviation, and range of the observations. The count variables were analyzed by using the *χ*^2^ or Fisher’s test, expressing as a number. A statistically significant difference was set at *p* < 0.05.

## Results

**Figure 2:**
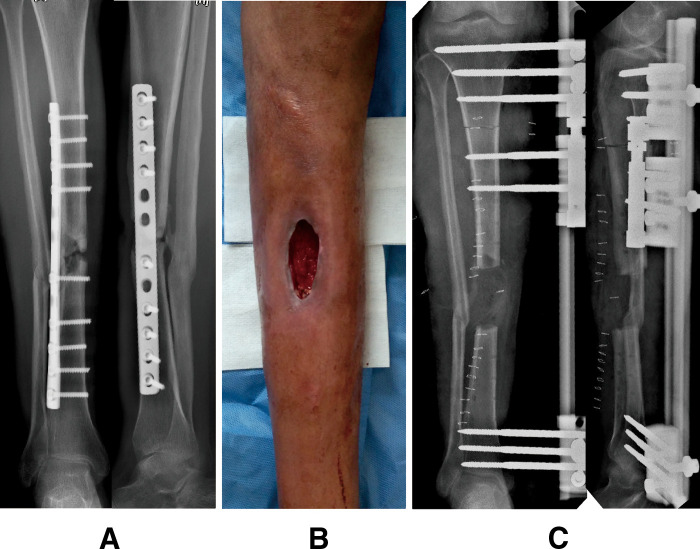
A 42-year-old man who suffered chronic osteomyelitis in his right tibia after internal fixation treatment followed by a road traffic accident and successfully treated by the bifocal bone transport (BFT) technique from proximal to distal. (**A**) Preoperative anteroposterior (AP) and lateral radiographs. (**B**) Preoperative general appearance, showing soft tissue defects with drainage and sinus. (**C**) AP and lateral radiographs immediately after radical debridement and installation of the monolateral external fixator; there were 6-cm bone defects. A BFT technique from proximal to distal was performed to reconstruct the injured limb.

**Figure 3:**
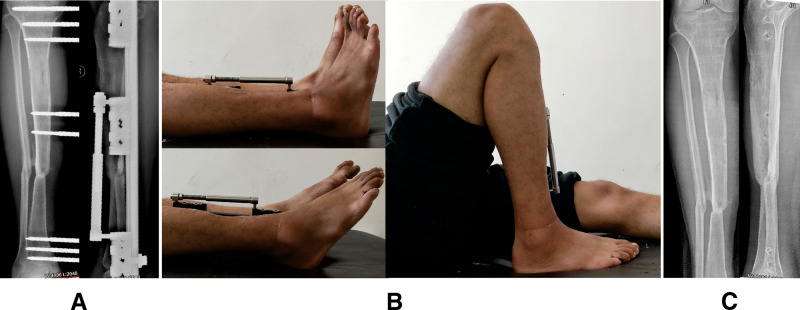
Images of the same patient shown in Figure 2. (**A**) Radiographs reveal the complete consolidation and docking site union. (**B**) General appearance before frame removal, showing the satisfactory range of motion of knee and ankle joint results. (**C**) Radiographs 6 months later after removing the external fixator.

**Figure 4:**
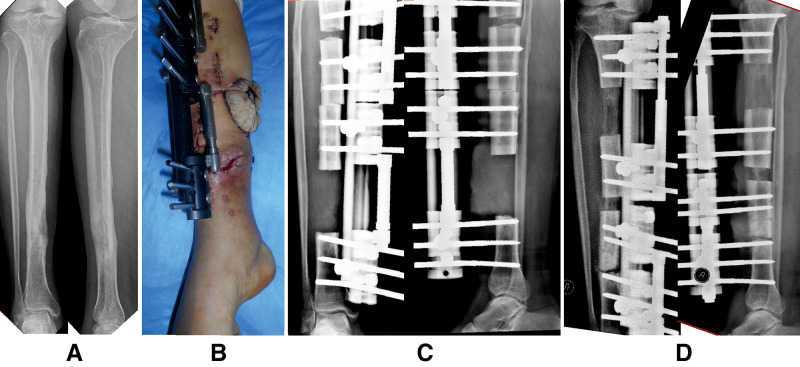
A 53-year-old man suffered chronic osteomyelitis in his right tibia after internal fixation treatment due to a crushing injury caused by a heavy object and was successfully managed by the trifocal bone transport technique (tandem transport, from proximal to distal). (**A**) Preoperative radiographs indicated that the infectious lesion was located at the distal one-third of the tibial shaft. (**B**) Removal of devitalized bone and soft tissue by radical debridement; the soft tissue defect was treated by using a local tissue flap. (**C**) There were 9-cm bone defects, and a trifocal tandem bone transport from proximal to distal was conducted for the limb reconstruction.

**Figure 5:**
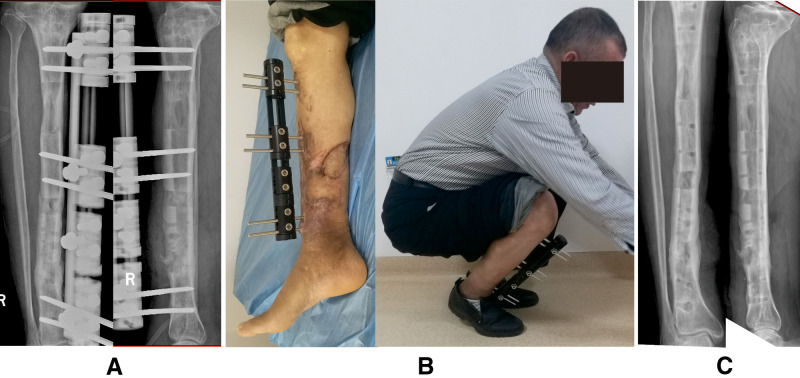
Images of the same patient shown in Figure 4. (**A**) Complete consolidation and docking site union after docking in 3 months. (**B**) Satisfactory functional recovery before monolateral external fixator removal. (**C**) Radiographs 9 months later after removing the frame.

There were no statistically significant differences in demographic data between the two groups (*p* > 0.05), except for the mean defect size (*p* < 0.05). The typical BFT is shown in Figures 2, 3, while TFT is exhibited in Figures 4, 5.

The mean OD was 122.5 ± 11.2 min in the BFT group, while it was 161.9 ± 8.9 min in the TFT group (*p* < 0.05). The DT in BFT was longer than that in TFT (96.8 ± 22.6 days, 65.9 ± 10.8 days, *p* < 0.05), as well as the EFT (474.5 ± 103.2 days, 328.0 ± 57.2 days, *p* < 0.05). All patients achieved an infection-free union finally. The average EFI in BFT (60.8 ± 1.9 days/cm) was larger than that in TFT (34.8 ± 2.1 days/cm) (*p* < 0.05). More details are given in Table 2.

**Table 1:** Demographics of the two groups.

Parameter	BFT group	TFT group	Statistical value	*p*-Value
Patients	32	21	–	–
Gender				
Male	23	16	0.121	0.727
Female	9	5		
Age (year)	38.2 ± 12.3	39.9 ± 12.9	−0.479	0.634
Etiology of bone defect				
Infected non-union	10	7	0.025	0.874
Chronic osteomyelitis	22	14		
Injured tibia				
Left	13	9	0.026	0.872
Right	19	12		
Location of bone defect				
Proximal	5	3	0.164	0.921
Middle	18	11		
Distal	9	7		
Mean previous operation time	2.5 ± 0.9	2.6 ± 1.1	−0.307	0.760
Mean defect size (cm)	7.8 ± 1.8	9.4 ± 1.5	−3.340	0.002

*BFT, bifocal bone transport; TFT, trifocal bone transport.*

**Table 2:** Comparison of the results of the two groups.

Parameter	BFT group	TFT group	Statistical value	*p*-Value
Mean OD (min)	122.5 ± 11.2	161.9 ± 8.9	−13.544	*p* < 0.001
Mean DT (days)	96.8 ± 22.6	65.9 ± 10.8	5.833	*p* < 0.001
Mean EFT (days)	474.5 ± 103.2	328.0 ± 57.2	5.919	*p* < 0.001
Mean EFI (days/cm)	60.8 ± 1.9	34.8 ± 2.1	46.492	*p* < 0.001

*BFT, bifocal bone transport; TFT, trifocal bone transport; OD, operation duration; DT, docking time; EFT, external fixation time; EFI, external fixation index.*

According to the ASAMI bone results, in the BFT group, 13 patients were in excellent condition, 15 were in good condition, 3 fair, and 1 poor. In the TFT group, 11 patients were in excellent condition, 8 in good condition, and 2 fair. The ASAMI functional results showed that, in the BFT group, 10 patients were in excellent condition, 16 were good, and 6 fair. In the TFT group, 8 patients were excellent, 10 good, and 3 fair. There was no significant difference between the two groups in both ASAMI bone and functional scores (*p* > 0.05) (Table 3).

**Table 3:** Results of ASAMI scores.

Parameter	Excellent	Good	Fair	Poor	Failure	*p*-Value
Bone results						
BFT group	13	15	3	1	–	0.903
TFT group	11	8	2	0	–	
Functional results						
BFT group	10	16	6	0	0	0.844
TFT group	8	10	3	0	0	

*BFT, bifocal bone transport; TFT, trifocal bone transport.*

*ASAMI criteria:*

*Bone results*

*Excellent: Union, no infection, deformity <7°, limb length discrepancy (LLD) <2.5 cm.*

*Good: Union plus any two of the following: absence of infection, deformity <7°, LLD <2.5 cm.*

*Fair: Union plus any one of the following: absence of infection, deformity <7°, LLD <2.5 cm.*

*Poor: Non-union/refracture/union plus infection plus deformity >7° plus LLD >2.5 cm.*

*Functional results*

*Excellent: Active, no limp, minimum stiffness (loss of <15° knee extension/<15° ankle dorsiflexion) no reflex sympathetic dystrophy (RSD), insignificant pain.*

*Good: Active, with one or two of the following: limb, stiffness, RSD, and significant pain.*

*Fair: Active, with three or all of the following: limb, stiffness, RSD, and significant pain.*

*Poor: Inactive (unemployment or the inability to return to daily activities because of injury).*

*Failure: Amputation.*

Based on Paley, there were 43 problems, 20 obstacles, and 31 complications in the BFT group, and 17 problems, 8 obstacles, and 11 complications were observed in the TFT group. The mean difficulties in BFT (2.9/patient) were larger than those in TFT (1.7/patient) (*p* < 0.05). Complications were more prevalent in the BFT group (31 complications of 32 patients) than in the TFT group (11 complications of 21 patients) (*p* < 0.05). More details are given in Tables 4, 5.

**Table 4:** Difficulties during treatment in the two groups.

Difficulty	BFT group (*n* = 32)	TFT group (*n* = 21)	*p*-Value
Problem	43	17	
Obstacle	20	8	
Complication	31	11	
Total difficulties	94	36	
Mean difficulties/patient	2.9	1.7	0.001

*BFT, bifocal bone transport; TFT, trifocal bone transport.*

**Table 5:** Summary of complications in the two groups.

Complications	BFT group (*n* = 32)		TFT group (*n* = 21)		*p*- Value
	Number	Percentage	Number	Percentage	
Pin tract infection	5	15.6	2	9.5	
Axial deviation	7	21.9	3	14.3	
Soft tissue incarceration	3	9.4	1	4.8	
Joint stiffness	11	34.4	5	23.8	
Delayed union	4	12.5	0	0.0	
Non-union	1	3.1	0	0.0	
Refracture	0	0.0	0	0.0	
Total complications	31		11		
Mean complications/ patient	1.0		0.5		0.032

*BFT, bifocal bone transport; TFT, trifocal bone transport*.

Five patients in BFT and two patients in TFT suffered deep pin tract infection and were successfully managed by screw replacement and intravenous antibiotics. The axial deviation was observed in seven cases in BFT and three cases in TFT. These patients underwent apparatus modification, docking site revision, and malalignment correction with regional anesthesia under image intensifier control. Three patients in BFT and one in TFT suffered soft tissue incarceration. Surgical intervention was performed to resect the interposed soft tissues, freshen the bone ends, and reopen the medullary canal. Joint stiffness was observed in 16 patients (11 in BFT and 5 in TFT) and successfully treated by a surgical release. Four patients in BFT suffered delayed union, and the “accordion maneuver” technique contributed to satisfactory outcomes. One patient developed non-union in the BFT group and finally achieved bone union by autogenous iliac crest bone grafting. No neurovascular injury, psychological problems, and refracture were observed in the 53 patients.

## Discussion

The ultimate way of treating bone defects is to reconstruct a fully functional extremity without any unacceptable deformities or limb length discrepancy (24). Undoubtedly, the treatment of a long segmental bone defect is a challenge for orthopedic surgeons, especially when it is accompanied by deep infection. It is necessary for most treating surgeons to manage bone defects and infection simultaneously. Options for the treatment of bone defects are varied (6–13), while the subsequent outcomes are not completely satisfactory. The concept of DO given by Ilizarov, in which bone regeneration occurs when it is subjected to tensile stress, contributes to managing this complex problem (28).

At present, the two common techniques for long bone defects are acute shortening followed by relengthening (AST) and bone transport using an external fixator. However, neurovascular injury, limb discrepancy, and blood circulation obstacles are problems worth considering when the AST is performed on patients with bone defects >5 cm (13, 20, 29). The bone transport technique has been widely applied to manage bone defects for decades (14, 16–20, 30), and problems, including infection, deformity, joint stiffness, or limb discrepancy, can be resolved simultaneously. The main drawback of the bone transport technique is the long procedure done in a cumbersome frame, resulting in inconveniencing the daily life of patients and producing numerous complications, including pin tract problems, joint stiffness, pain, and psychological symptoms. These problems have been the main obstacles to the extended application of bone transport. In addition, many patients with bone defects caused by an infection usually undergo several previous operations that fail and develop compromised surrounding soft tissues. Therefore, a treatment of shorter duration and fewer complications is really a goal worth striving for.

Several methods have been developed to reduce the EFI, reduce the potential complications, and achieve satisfactory clinical results. Gupta et al. (31) performed a three-stage treatment in 14 consecutive patients with tibial-infected non-union using a long lateral locked plate and a six-pin monorail fixator. In this group of patients, the mean defect size was 6.4 cm and the mean external fixator index was 21.2 days/cm, while the complication rate was 0.5 per patient. The researchers declared that composite fixation reduces the fixator time and the associated complications for patients with a segmental tibial defect due to an infected non-union and provides high emotional acceptance of final clinical outcomes. Gulabi et al. (32) developed a technique to reduce complications by using a circular external fixator combined with an intramedullary nail to achieve union, limb lengthening, and regenerate stability. In their treatment of five tibial non-union patients with bone defects, they acquired satisfactory outcomes in all cases. Similarly, Kocaoglu et al. (26) also summarized their experience with DO in the management of bone defects and limb shortening due to a radical debridement of chronic osteomyelitis using an external fixator combined with an intramedullary nail. Although the mentioned techniques involve less duration in the external fixator, their combination with a nail cannot accelerate regenerate consolidation and resolve chronic infection simultaneously. Furthermore, the external treatment, combined with the internal fixation treatment, requires several additional surgical interventions. It is difficult for most patients to accept this combined treatment due to their unpleasant experiences in numerous previous operations.

The TFT technique was proposed to accelerate the defect closure and shorten the treatment duration. Considering the biological characteristic of incapable speed up lengthening in just one osteotomy site, the TFT was derived from the theoretical basis of distraction simultaneously using two osteotomy leads to effectively double lengthening speed. In this procedure, the regeneration time is theoretically decreased to 50%, and the total treating time is reduced accordingly (24). Additionally, the regenerate consolidation time is inversely proportional to the bone defect length, and two shorter fragments are, therefore, capable of rapid consolidation compared with only one fragment.

Borzunov (21) performed an experimental comparison in the treatment of tibial defects by first using the BFT and multilevel techniques and subsequently extending them to clinical application. For the management of patients with large tibial defects that ranged from 12 to 14 cm using multilevel techniques, distraction duration could be reduced by 2.5 times, and the fixation period could be reduced between 1.3 and 1.9 times. Sala et al. (33) compared the clinical outcomes of TFT and BFT in the treatment of postinfectious segmental tibial bone defects with a combined Ilizarov and Taylor spatial frame method. In their retrospective study of 12 patients with atrophic tibial non-unions, although the average lengthening size was increased (9.7 cm in TFT, 5.5 cm in BFT) in the TFT group, there was a shorter mean period in the frame (379 days in TFT, 457 in BFT). In addition, the TFT technique reduced the mean lengthening index (1.31 months/cm in TFT, 2.63 months/cm in BFT).

In the present study, the clinical results of 53 patients with long segmental tibial bone defects treated by the bifocal or TFT technique were retrospectively analyzed and compared. The mean defect size was significantly increased in the TFT group (9.4 ± 1.5 cm in TFT, 7.8 ± 1.8 cm in BFT). In contrast, the mean DT (65.9 ± 10.8 days in TFT, 96.8 ± 22.6 days in BFT), the mean EFT (328.0 ± 57.2 days in TFT, 474.5 ± 103.2 days in BFT), and the mean EFI (34.8 ± 2.1 days/cm in TFT, 60.8 ± 1.9 days/cm in BFT) were significantly reduced. The results were comparable to those of the previous studies (21, 33).

A previous study reported that hypoplastic bone formation was a common complication when bone defects exceed 5 cm or 40% of the injured bone when treated by BFT (27). The regenerate consolidation will be affected by the osteotomy technique and the location, distraction length, and blood supply of the transported fragment. In our study, there were four patients who suffered delayed consolidation in the BFT group, but none was observed in the TFT group. Based on our experience, we recommend the low-energy osteotomy technique, timely lengthening speed adjustment, and the application of the TFT technique in massive bone defects for avoiding the problem of delayed consolidation.

Although additional surgical procedures of one transport sliding block and an osteotomy site in TFT may increase the complications corresponding to Schanz screws and the distraction zone, faster regeneration and early frame removal have counteracted this negative effect. In this study, the BFT group showed a statistically significant increase in the mean rate of difficulty (2.9 difficulties/patient in BFT and 1.7 difficulties/patient in TFT) and complication (1.0 complications/patient in BFT and 0.5 complications/patient in TFT), and this can be explained by the longer frame duration in these patients. The only shortcoming in the TFT group was the significantly increased operation time (161.9 ± 8.9 min in TFT, 122.5 ± 11.2 min in BFT).

Furthermore, compared with previous studies (17, 29, 33), the Ilizarov circular external fixator is relatively cumbersome, complex, difficult to learn, time-consuming, and fraught with a lot of potential complications. The monolateral rail external fixator used in this study, working on the same principles as the Ilizarov circular external fixator, is portable, easy to construct, and has a short learning curve. It is also easy to adjust the sliding clamps adapted to the transporting bone fragments without altering the nut bolts over the threaded rods, unlike in the Ilizarov circular external fixator.

The clinical results of the present study reveal that both BFT and TFT achieve satisfactory outcomes in the reconstruction of long segmental tibial bone defects caused by infection using a monolateral rail external fixator. Although there were no significant differences between BFT and TFT in the final bone and functional results, the EFI, difficulties, and complications were significantly decreased in the TFT group. Our experience suggests that the most vital step is radical debridement of the infectious tissues in the reconstruction of bone defects caused by infection. In addition, a comprehensive understanding of frames, prudent patient selection, appropriate pin insertion, meticulous care, early detection of complications, and proper intervention or psychological counseling all ensure satisfactory results.

The present study may be limited by its retrospective nature with only a single-center small sample size, and, therefore, a conservative attitude should be adopted while making interpretations of our results. Multicentered trials with larger sample sizes, life quality assessments, and mental evaluations should be adopted in future investigations.

## Conclusion

Both the trifocal and the BFT techniques achieve satisfactory clinical outcomes in the reconstruction of long segmental tibial bone defects caused by infection using a monolateral rail external fixator. The TFT technique can significantly decrease the DT, EFT, EFI, difficulties, and complications compared with the BFT technique.

## Data Availability

The original contributions presented in the study are included in the article/supplementary material; further inquiries can be directed to the corresponding author/s.

## References

[1] Reichert JC Saifzadeh S Wullschleger ME Epari DR Schütz MA Duda GN The challenge of establishing preclinical models for segmental bone defect research. Biomaterials. (2009) 30:2149–63. 10.1016/j.biomaterials.2008.12.050 19211141

[2] Sadek AF Laklok MA Fouly EH Elshafie M. Two stage reconstruction versus bone transport in management of resistant infected tibial diaphyseal nonunion with a gap. Arch Orthop Trauma Surg. (2016) 136:1233–41. 10.1007/s00402-016-2523-8 27447880

[3] Azzam W Atef A. Our experience in the management of segmental bone defects caused by gunshots. Int Orthop. (2016) 40:233–8. 10.1007/s00264-015-2870-z 26152244

[4] Decoster TA Gehlert RJ Mikola EA Pirela-Cruz MA. Management of posttraumatic segmental bone defects. J Am Acad Orthop Surg. (2004) 12:28–38. 10.5435/00124635-200401000-00005 14753795

[5] Eralp L Kocaoglu M Rashid H. Reconstruction of segmental bone defects due to chronic osteomyelitis with use of an external fixator and an intramedullary nail. Surgical technique. J Bone Joint Surg Am. (2007) 89(Suppl 2 Pt.2):183–95. 17768214 10.2106/JBJS.G.00306

[6] Friedrich JB Moran SL Bishop AT Wood CM Shin AY. Free vascularized fibular graft salvage of complications of long-bone allograft after tumor reconstruction. J Bone Joint Surg Am. (2008) 90:93–100. 10.2106/JBJS.G.00551 18171962

[7] Germain MA Mascard E Dubousset J Nguefack M. Free vascularized fibula and reconstruction of long bones in the child–our evolution. Microsurgery. (2007) 27:415–9. 10.1002/micr.20384 17596859

[8] Masquelet AC Begue T. The concept of induced membrane for reconstruction of long bone defects. Orthop Clin North Am. (2010) 41:27–37. 10.1016/j.ocl.2009.07.011 19931050

[9] Morris R Hossain M Evans A Pallister I. Induced membrane technique for treating tibial defects gives mixed results. Bone Joint J. (2017) 99-B:680–5. 10.1302/0301-620X.99B5.BJJ-2016-0694.R2 28455479

[10] Pipitone PS Rehman S. Management of traumatic bone loss in the lower extremity. Orthop Clin North Am. (2014) 45:469–82. 10.1016/j.ocl.2014.06.008 25199419

[11] Pirela-Cruz MA Decoster TA. Vascularized bone grafts. Orthopedics. (1994) 17:407–12. 10.3928/0147-7447-19940501-05 8036184

[12] Szabo RM Anderson KA Chen JL. Functional outcome of en bloc excision and osteoarticular allograft replacement with the Sauve-Kapandji procedure for Campanacci grade 3 giant-cell tumor of the distal radius. J Hand Surg Am. (2006) 31:1340–8. 10.1016/j.jhsa.2006.06.004 17027797

[13] Tetsworth K Paley D Sen C Jaffe M Maar DC Glatt V Bone transport versus acute shortening for the management of infected tibial non-unions with bone defects. Injury. (2017) 48:2276–84. 10.1016/j.injury.2017.07.018 28734494

[14] Aktuglu K Erol K Vahabi A. Ilizarov bone transport and treatment of critical-sized tibial bone defects: a narrative review. J Orthop Traumatol. (2019) 20:22. 10.1186/s10195-019-0527-1 30993461 PMC6468024

[15] Aktuglu K Gunay H Alakbarov J. Monofocal bone transport technique for bone defects greater than 5 cm in tibia: our experience in a case series of 24 patients. Injury. (2016) 47(Suppl 6):S40–6. 10.1016/S0020-1383(16)30838-5 28040086

[16] Baumgart R Schuster B Baumgart T. Callus distraction and bone transport in the treatment of bone defects. Orthopade. (2017) 46:673–80. 10.1007/s00132-017-3441-3 28725933

[17] Catagni MA Azzam W Guerreschi F Lovisetti L Poli P Khan MS Trifocal versus bifocal bone transport in treatment of long segmental tibial bone defects. Bone Joint J. (2019) 101-B:162–9. 10.1302/0301-620X.101B2.BJJ-2018-0340.R2 30700126

[18] Jiang Q Huang K Liu Y Chi G. Using the Ilizarov technique to treat limb shortening after replantation of a severed lower limb: a case report. Ann Transl Med. (2020) 8:1025. 10.21037/atm-20-5316 32953825 PMC7475505

[19] Vesely R Prochazka V. Callus distraction in the treatment of post-traumatic defects of the femur and tibia. Acta Chir Orthop Traumatol Cechoslov. (2016) 83:388–92. 28026734

[20] Wu Y Yin Q Rui Y Sun Z Gu S. Ilizarov technique: bone transport versus bone shortening-lengthening for tibial bone and soft-tissue defects. J Orthop Sci. (2018) 23:341–5. 10.1016/j.jos.2017.12.002 29290472

[21] Borzunov DY. Long bone reconstruction using multilevel lengthening of bone defect fragments. Int Orthop. (2012) 36:1695–700. 10.1007/s00264-012-1562-1 22581353 PMC3535043

[22] Yushan M Ren P Abula A Alike Y Abulaiti A Ma C Bifocal or trifocal (double-level) bone transport using unilateral rail system in the treatment of large tibial defects caused by infection: a retrospective study. Orthop Surg. (2020) 12:184–93. 10.1111/os.12604 31943836 PMC7031621

[23] Zhang Y Wang Y Di J Peng A. Double-level bone transport for large post-traumatic tibial bone defects: a single centre experience of sixteen cases. Int Orthop. (2018) 42:1157–64. 10.1007/s00264-017-3684-y 29129017

[24] Paley D Catagni MA Argnani F Villa A Benedetti GB Cattaneo R. Ilizarov treatment of tibial nonunions with bone loss. Clin Orthop Relat Res. (1989):146–65. 2924458

[25] Paley D. Problems, obstacles, and complications of limb lengthening by the Ilizarov technique. Clin Orthop Relat Res. (1990):81–104. 2403498

[26] Kocaoglu M Eralp L Rashid HU Sen C Bilsel K. Reconstruction of segmental bone defects due to chronic osteomyelitis with use of an external fixator and an intramedullary nail. J Bone Joint Surg Am. (2006) 88:2137–45. 10.2106/JBJS.E.01152 17015589

[27] Borzunov DY Chevardin AV. Ilizarov non-free bone plasty for extensive tibial defects. Int Orthop. (2013) 37:709–14. 10.1007/s00264-013-1799-3 23377109 PMC3609969

[28] Ilizarov GA. The tension-stress effect on the genesis and growth of tissues. Part I. The influence of stability of fixation and soft-tissue preservation. Clin Orthop Relat Res. (1989):249–81. 10.1097/00003086-198901000-00038 2910611

[29] Mahaluxmivala J Nadarajah R Allen PW Hill RA. Ilizarov external fixator: acute shortening and lengthening versus bone transport in the management of tibial non-unions. Injury. (2005) 36:662–8. 10.1016/j.injury.2004.10.027 15826629

[30] Biz C Crimi A Fantoni I Vigo M Iacobellis C Ruggieri P. Functional outcome and complications after treatment of comminuted tibial fractures or deformities using Ilizarov bone transport: a single-center study at 15- to 30-year follow-up. Arch Orthop Trauma Surg. (2021) 141:1825–33. 10.1007/s00402-020-03562-9 32734449 PMC8497293

[31] Gupta S Malhotra A Mittal N Garg SK Jindal R Kansay R. The management of infected nonunion of tibia with a segmental defect using simultaneous fixation with a monorail fixator and a locked plate. Bone Joint J. (2018) 100-B:1094–9. 10.1302/0301-620X.100B8.BJJ-2017-1442.R1 30062945

[32] Gulabi D Erdem M Cecen GS Avci CC Saglam N Saglam F. Ilizarov fixator combined with an intramedullary nail for tibial nonunions with bone loss: is it effective? Clin Orthop Relat Res. (2014) 472:3892–901. 10.1007/s11999-014-3640-8 24777722 PMC4397756

[33] Sala F Thabet AM Castelli F Miller AN Capitani D Lovisetti G Bone transport for postinfectious segmental tibial bone defects with a combined ilizarov/taylor spatial frame technique. J Orthop Trauma. (2011) 25:162–8. 10.1097/BOT.0b013e3181e5e160 21321507

